# Supplementary material for: Dynamic transcriptomic profiles of zebrafish gills in response to zinc supplementation
Source: BMC Genomics. 2010 Oct 11;11:553. doi: 10.1186/1471-2164-11-553 (PMC3091702; doi:10.1186/1471-2164-11-553)
Supplement: Additional file 2 — Interactive Direct Interaction Network representing the molecular interactions between zinc, copper, iron, calcium and proteins encoded by transcripts changed by zinc supplementation. Mini web-site containing index.html and hyperlinked pages in subdirectory describing a Direct Interaction Network automatically generated based on curated interactions contained within the proprietary PathwayArchitect database. Ovals represent proteins and the circles symbolize metal ions. Objects are coloured by their abundance in zebrafish at the time-point they were significantly different from the control is a scale from -4 fold (dark green) to +4 fold (dark red). Where significant differences were found at more than one time-point, the colour overlay shows expression at the first instance. Dark blue squares denote 'binding', and light blue squares 'expression'; green squares stand for 'regulation', green diamonds for 'metabolism', and green circles for 'promoter binding'. Arrow heads indicate directionality of the interaction where annotated. All nodes and edges can be further interrogated by selecting the relative area of the image. [file 1471-2164-11-553-S2.zip › PathwayArchitect Zn xs DIN/596355.html]

# TRANSPORT:

|  |  |
| --- | --- |
| Type | TRANSPORT |
| Effect | None |


---

|  |  |
| --- | --- |
| Score | 0 |


---

|  |  |
| --- | --- |
| Reference Count | 8 |


---

|  |  |
| --- | --- |
| Mechanism | Unknown |


---

|  |  |
| --- | --- |
| Reference:0 || Sentence | "These findings demonstrate that during the late hypodynamic phase of sepsis, the observed decrease in myocardial contractility was due to the decrease in phospholamban phosphorylation, which resulted in decreased Ca2+ transport across the SR." |
| PMID | 12022759 |
| Year | 2002 |
| Species | Rat |
| Journal | Shock |
| RefScore | 0 |
| Source | PArchNLP |
  |
|  |  |


---

|  |  |
| --- | --- |
 Reference:1 || Sentence | "Wild-type phospholamban, as well as phospholamban with different point mutations in the transmembrane region, inhibited both Ca2+ transport and ATP hydrolysis by the recombinant Ca2+ pump." |
| PMID | 10603939 |
| Year | 1998 |
| Journal | Ann N Y Acad Sci |
| RefScore | 1 |
| Source | PArchNLP |
  ||  |  |


---

|  |  |
| --- | --- |
 Reference:2 || Sentence | "A role for phospholamban in the control of Ca2+ transport by the sarcoplasmic reticulum has been postulated, but the mechanism is incompletely understood." |
| PMID | 2848034 |
| Year | 1988 |
| Journal | J Biol Chem |
| RefScore | 1 |
| Source | PArchNLP |
  ||  |  |


---

|  |  |
| --- | --- |
 Reference:3 || Sentence | "Cardiac sarcoplasmic reticulum contains a low-molecular-weight protein, phospholamban, the phosphorylation of which is required for Ca2+ transport." |
| PMID | 7023251 |
| Year | 1981 |
| Journal | Am J Physiol |
| RefScore | 2 |
| Source | PArchNLP |
  ||  |  |


---

|  |  |
| --- | --- |
 Reference:4 || Sentence | "A monoclonal antibody that recognized amino acid residues 9-17 of PLB had no significant effect on Ca2+ transport by AT-1 SR vesicles, but it increased Ca2+ transport into mouse ventricular SR vesicles greater than 10-fold by reversing PLB inhibition of SERCA 2 at low ionized Ca2+ concentration." |
| PMID | 8505284 |
| Year | 1993 |
| Species | Mouse |
| Journal | J Biol Chem |
| RefScore | 3 |
| Source | PArchNLP |
  ||  |  |


---

|  |  |
| --- | --- |
 Reference:5 || Sentence | "Based on these results, we conclude that the cytoplasmic domain of PLB, containing the phosphorylation sites, by itself is insufficient to inhibit the Ca2+ pump and that the transmembrane region, which stabilizes the pentamer, is also essential for Ca2+ transport regulation." |
| PMID | 8505284 |
| Year | 1993 |
| Species | Mouse |
| Journal | J Biol Chem |
| RefScore | 2 |
| Source | PArchNLP |
  ||  |  |


---

|  |  |
| --- | --- |
 Reference:6 || Sentence | "The correlation between phospholamban and sarcoplasmic reticulum Ca(2+)-transporting ATPase levels and the magnitude of phospholamban-mediated stimulation of sarcoplasmic reticulum Ca2+ transport was examined in microsomes prepared from rabbit and canine cardiac, slow twitch and fast twitch skeletal muscle." |
| PMID | 1345340 |
| Year | 1992 |
| Journal | Second Messengers Phosphoproteins |
| RefScore | 0 |
| Source | PArchNLP |
  ||  |  |


---

|  |  |
| --- | --- |
 Reference:7 || Sentence | "Sarcoplasmic reticulum Ca2+ transport assays indicated similar decreases in SERCA2 Ca2+ affinity by mutant or wild-type phospholamban." |
| PMID | 9837953 |
| Year | 1998 |
| Species | Mouse |
| Journal | J Biol Chem |
| RefScore | 1 |
| Source | PArchNLP |
  |


---

|  |  |
| --- | --- |
